# Editorial: Hair Bundles—Development, Maintenance, and Function

**DOI:** 10.3389/fcell.2021.800410

**Published:** 2021-11-15

**Authors:** Zhigang Xu, Anthony W. Peng, Wei Xiong

**Affiliations:** ^1^ Shandong Provincial Key Laboratory of Animal Cell and Developmental Biology, School of Life Sciences, Shandong University, Qingdao, China; ^2^ Department of Physiology and Biophysics, University of Colorado Anschutz Medical Campus, Aurora, CO, United States; ^3^ School of Life Sciences, IDG/McGovern Institute for Brain Research at Tsinghua, Tsinghua University, Beijing, China

**Keywords:** hair cells, hair bundles, stereocilia, kinocilium, mechano-electrical transduction

Hair bundles are hairy-looking cell protrusions at the apical surface of mechanosensitive hair cells, consisting of hundreds of actin-based stereocilia and one microtubule-based kinocilium in each cell (Flock and Cheung, 1977). The kinocilium is important for hair bundle development as well as planar cell polarity (PCP) establishment, while the stereocilia are indispensable for mechano-electrical transduction (MET) (Hudspeth and Jacobs, 1979; Jones et al., 2008). The tightly-regulated process of hair bundle development started to be appreciated after Lew Tilney’s seminal work (Tilney et al., 1992). With the rapid progress of genetic, proteomic, and microscopic techniques, many proteins have been identified to play important roles in the development, maintenance, and/or function of hair bundles (Barr-Gillespie, 2015; McGrath et al., 2017; Velez-Ortega and Frolenkov, 2019). Nevertheless, a lot more remain unknown about this fantastic subcellular structure responsible for sound and balance detection.

In the present Research Topic, Landin Malt et al. show that non-canonical Wnt signaling regulates cochlear outgrowth and PCP through Gsk3β inhibition. Zhang et al. investigate the role of RNA-binding protein RBM24 in hair cell development, and show that Rbm24a deficiency in zebrafish affects development of hair cells as well as stereocilia through regulating the stability of its downstream target mRNAs. Li et al. show that GRXCR2 interacts with CLIC5 at the base of stereocilia and plays an essential role in stereocilia development. Kannan-Sundhari et al. reveal that bromodomain protein BRD4 is essential for stereocilia development as well as hair cell function, and Liu et al. show that lysyl oxidase LOXL3 inactivation leads to hair cell deficits including stereocilia disorganization. Wang and Zhou review the role of kinocilia in the development of hair cells and related diseases, while Cirilo et al. review the functional role of class III myosins in the stereocilia. Together, these works help us to learn more about the underlying mechanism of hair bundle development and maintenance.

The mechanic stimuli such as sound deflect hair bundles, then open the MET channels localized at the tips of shorter row stereocilia, resulting in influx of cations into the hair cells (Hudspeth and Jacobs, 1979; Beurg et al., 2009). Peng et al. show that hair bundles stimulated with a fluid jet exhibit spatial non-uniformities and two viscoelastic-like mechanisms, which are important for us to understand the mechanics of the mammalian hair bundles. TMC1/TMC2 have been suggested to be the pore-forming components of MET channels (Pan et al., 2018; Jia et al., 2020). In the zebrafish, there are one Tmc1 and two Tmc2 orthologs, and Zhu et al. show that Tmc reliance is biased by the hair cell subtype and position in the zebrafish inner ear. These works shed light on the mechanism of how stereocilia respond to mechanical stimuli and result in MET.

Our understanding of the hair bundles cannot improve without technical advances. Electron microscopy (EM) has been extensively used to understand the development, maintenance, and function of hair bundles. Ivanchenko et al. systematically summarize different EM techniques commonly used in studying hair bundles such as scanning electron microscopy (SEM), transmission electron microscopy (TEM), and focused-ion-beam scanning electron microscopy (FIB-SEM), and provide detailed protocols for sample preparation, treatment, and imaging. Miller et al. compare stereocilia measurements from living tissue, tissue mildly-fixed for fluorescent imaging, or tissue strongly-fixed for SEM, and illustrate the requirement of high accuracy to understand hair bundle mechanotransduction. Lin et al. turn to structural biology and reveal that the harmonin homology domain (HHD) of PDZD7 binds lipid and mediates the localization of PDZD7 to the plasma membrane, therefore provide mechanistic explanations for human deafness-associated mutations in PDZD7.

In the present Research Topic, researchers also show some other interesting findings. Li et al. show that albeit specially expressed in the inner ear hair cells and localized at the tips of stereocilia, annexin A4 (ANXA4) is dispensable for development and function of stereocilia and hair cells. Moreover, Gilbert et al. show that actin crosslinking family protein 7 (ACF7) is localized to the cuticular plate and the circumferential band in hair cells, but its inactivation does not have a significant effect on the hair bundle or hair cells. These studies suggest that not all hair cell-specific or hair bundle-enriched proteins are essential for hair cells to develop or function. Nevertheless, possibilities remain that these proteins are required for hair cell processes that were not examined in these studies.

In summary, this Research Topic presents some recent advances in the development, maintenance, and function of hair bundles. However, despite the rapid progress in this field, there is much more to learn. For example, how are the heights of stereocilia in different rows precisely controlled? How are the stereocilia row numbers determined? How is the MET machinery organized and regulated? We hope that we can have answers for these questions in the near future.
